# Rapid End-Point Quantitation of Prion Seeding Activity with Sensitivity Comparable to Bioassays

**DOI:** 10.1371/journal.ppat.1001217

**Published:** 2010-12-02

**Authors:** Jason M. Wilham, Christina D. Orrú, Richard A. Bessen, Ryuichiro Atarashi, Kazunori Sano, Brent Race, Kimberly D. Meade-White, Lara M. Taubner, Andrew Timmes, Byron Caughey

**Affiliations:** 1 Laboratory of Persistent Viral Diseases, Rocky Mountain Laboratories, National Institute of Allergy and Infectious Disease, Hamilton, Montana, United States of America; 2 Department of Biomedical Sciences and Technologies, University of Cagliari, Monserrato, Italy; 3 Veterinary Molecular Biology, Montana State University, Bozeman, Montana, United States of America; 4 Department of Molecular Microbiology and Immunology, Nagasaki University Graduate School of Biomedical Sciences, Nagasaki, Kyushu, Japan; University of Alberta, Canada

## Abstract

A major problem for the effective diagnosis and management of prion diseases is the lack of rapid high-throughput assays to measure low levels of prions. Such measurements have typically required prolonged bioassays in animals. Highly sensitive, but generally non-quantitative, prion detection methods have been developed based on prions' ability to seed the conversion of normally soluble protease-sensitive forms of prion protein to protease-resistant and/or amyloid fibrillar forms. Here we describe an approach for estimating the relative amount of prions using a new prion seeding assay called real-time quaking induced conversion assay (RT-QuIC). The underlying reaction blends aspects of the previously described quaking-induced conversion (QuIC) and amyloid seeding assay (ASA) methods and involves prion-seeded conversion of the alpha helix-rich form of bacterially expressed recombinant PrP^C^ to a beta sheet-rich amyloid fibrillar form. The RT-QuIC is as sensitive as the animal bioassay, but can be accomplished in 2 days or less. Analogous to end-point dilution animal bioassays, this approach involves testing of serial dilutions of samples and statistically estimating the seeding dose (SD) giving positive responses in 50% of replicate reactions (SD_50_). Brain tissue from 263K scrapie-affected hamsters gave SD_50_ values of 10^11^-10^12^/g, making the RT-QuIC similar in sensitivity to end-point dilution bioassays. Analysis of bioassay-positive nasal lavages from hamsters affected with transmissible mink encephalopathy gave SD_50_ values of 10^3.5^–10^5.7^/ml, showing that nasal cavities release substantial prion infectivity that can be rapidly detected. Cerebral spinal fluid from 263K scrapie-affected hamsters contained prion SD_50_ values of 10^2.0^–10^2.9^/ml. RT-QuIC assay also discriminated deer chronic wasting disease and sheep scrapie brain samples from normal control samples. In principle, end-point dilution quantitation can be applied to many types of prion and amyloid seeding assays. End point dilution RT-QuIC provides a sensitive, rapid, quantitative, and high throughput assay of prion seeding activity.

## Author Summary

Prion diseases are deadly infectious neurodegenerative disorders of mammals which involve the misfolding of host prion protein. To better manage these diseases, we need to be able to detect and quantify the infectious particles, or prions, in biological samples. However, current tests lack the sensitivity, speed and/or quantitative capabilities required for many important applications in medicine, agriculture, wildlife biology and research. To address this problem, we have developed a new prion assay that is highly sensitive, rapid, and quantitative. This assay takes advantage of the ability of miniscule amounts of infectious prions to seed the misfolding of large excesses of normal prion protein in test tube reactions. Quantitation is achieved by testing a range of sample dilutions and determining loss of seeding activity, i.e. the end-point dilution. Similar analyses have long been used to quantify prions by inoculation into animals; however, such bioassays take months or years to perform and are both animal-intensive and expensive. Our new method provides a more practical means of detecting and quantifying prions. So far, we have applied this assay to prions from sheep, deer, and hamsters, and have found surprisingly high levels of prions in the nasal and cerebral spinal fluids of infected hamsters.

## Introduction

The transmissible spongiform encephalopathies (TSEs) or prion diseases are fatal neurodegenerative disorders that include human Creutzfeldt-Jakob disease (CJD), bovine spongiform encephalopathy (BSE), sheep scrapie, cervid chronic wasting disease (CWD), and transmissible mink encephalopathy (TME). The infectious agent, or prion, of the TSEs appears to be composed primarily of an abnormal, misfolded, oligomeric form of prion protein (PrP^Sc^). PrP^Sc^ is formed post-translationally from the normal cellular prion protein (PrP^C^) [1], [2]. PrP^Sc^, which in purified form can resemble amyloid fibrils, induces the polymerization and conformational conversion of PrP^C^ to infectious PrP^Sc^ [3]–[5] or to PrP^Sc^-like partially protease-resistant forms (PrP^res^) in a variety of *in vitro* reactions [4], [6]–[8]. These studies demonstrate that PrP^Sc^ can self-propagate, and although the mechanism is not fully understood, it appears to be a seeded or templated polymerization [9]–[11].

The ability to detect prions rapidly and sensitively would be an important asset in managing TSEs. Early prion detection in individuals is critical to the prevention of spread and the initiation of potential treatments. Prions can be found in a wide variety of tissues and accessible bodily fluids from infected mammalian hosts, including blood [12]–[17], breast milk [18], [19], saliva [15], [20], urine [21], [22], feces [23], and nasal fluids [24]. In most cases, our ability to rapidly measure prion infectivity in these fluids is limited by the low amount of infectious agent. Knowledge of the prion titers in these fluids or tissues and their products is key for prion diagnosis and in assessing the public health exposure risks to those materials.

The most direct and reliable assay for the detection of TSE infectivity is animal bioassay. Quantitation of infectivity can be achieved by end-point [25] or limiting dilution bioassays [26]. For some combinations of prion agent and host species, strong correlations between infectivity titer and disease incubation period have been established in laboratory rodents, allowing the use of incubation period to measure infectivity levels [27], [28]. The disadvantage of these bioassays is that they are animal-intensive, time-consuming and expensive. For certain murine-adapted scrapie strains, the cell culture based standard scrapie cell assay (SSCA) can also be used to measure infectivity levels by end-point and limiting dilution methods [29]. The SSCA offers several advantages over animal bioassays but it still requires weeks to perform and has been limited to a few mouse-adapted scrapie strains. The limitations of the animal bioassay and SSCA have provided strong motivation to develop more practical assays for prion quantitation.

Several extremely sensitive *in vitro* methods for prion detection have been reported. Using the protein misfolding cyclic amplification (PMCA) reaction in multi-round sonicated reactions with brain-derived PrP^C^ as a substrate, as little as 1 ag of PrP^sc^ can be detected [30]. The speed and practicality of PMCA assays have been improved by the use of bacterially-expressed recombinant PrP^C^ (rPrP^c^) [31] and by substituting shaking for the sonication step as described for quaking-induced conversion (QuIC) reactions [32], [33]. The QuIC assay can detect sub-femtogram amounts of PrP^Sc^ (less than one lethal intracerebral dose) in hamster brain homogenates (BH) within a single day. The effectiveness of the QuIC assay for prion detection was demonstrated by its ability to discriminate normal from prion-infected hamsters using 2 µl samples of cerebral spinal fluid (CSF) [32], [33] or nasal lavage [24]. The latest adaptations of QuIC reactions have led to the sensitive detection of variant CJD (vCJD) in human tissue and scrapie in sheep tissue [33]. The readout for QuIC and PMCA assays is the detection of specific protease-resistant prion-seeded products by immunoblotting, which is difficult to adapt to automated high-throughput formats. An alternative, and potentially higher-throughput approach was used for the amyloid seeding assay (ASA) in which the fluorescent dye thioflavin T (ThT) was used to detect prion seeding of rPrP^C^ polymerization [34]. The ASA can also detect protease sensitive disease-causing prions and has a 98% correlation with neuropathological signs of prion disease [35]. However, a potentially confounding aspect of ASA is the frequent spontaneous formation of rPrP fibrils (without seeding by prions) soon after prion-seeded reactions [34].

Until very recently, a major limitation of the PMCA, QuIC and ASA methods was the lack of prion quantitation. While the present paper was under review, Chen and colleagues reported a method called quantitative PMCA (qPMCA) in which PrP^Sc^ content is estimated by the number of PMCA rounds necessary for a positive response [36]. Here we describe a distinct end-point dilution approach to relative prion quantitation with *in vitro* prion seeding assays which is analogous to the end-point dilution titrations classically used in animal bioassays. At the same time, we describe a new prion-seeded rPrP^c^ polymerization assay, real-time (RT)-QuIC, which combines several aspects of the QuIC assay (intermittent shaking, rPrP^C^ preparation, sample preparation, and a lack of chaotropic salts) with a fluorescent ThT readout, but with greatly reduced spontaneous rPrP fibril formation. Some elements of this new assay were first developed using human rPrP^c^ and designated “real-time QUIC” by analogy with real-time PCR (R. Atarashi, K. Satoh, K. Sano, T. Fuse, N. Yamaguchi, D. Ishibashi, T. Matsubara, T. Nakagaki, H. Yamanaka, S. Shirabe, M. Yamada, H. Mizusawa, T. Kitamoto, G. Klug, A. McGlade, S. J. Collins, and N. Nishida, manuscript submitted). The latter manuscript describes the non-quantitative application of RT-QuIC to the detection of prions in CSF of human patients with multiple types of sporadic CJD. In the present study, we have applied RT-QuIC to prions of sheep, deer and hamsters, and measured prion seeding activity in the nasal fluids and CSF of prion-infected hamsters. In conjunction with the end-point dilution analysis that we describe here, the RT-QuIC assay can rapidly determine relative prion concentrations with a sensitivity that rivals that of animal bioassays, but with greatly reduced time and cost.

## Materials and Methods

### Recombinant prion protein purification

DNA Sequences coding for hamster (residues 90–231 and 23–231; accession K02234), deer (residues 24–234; accession AF156185) and sheep (residues 25–234; accession AJ567988) rPrP^c^ residues were amplified and ligated into the pET41 vector (EMD Biosciences) and sequences were verified. After transforming the plasmids into *E. coli* Rosetta cells (EMD Biosciences), we expressed the rPrP^c^ using the Overnight Express Autoinduction system (EMD Biosciences). Cell pellets from 0.25 L cultures were then put through two liquid nitrogen freeze thaw cycles and further lysed with BugBuster master mix (EMD Biosciences) to isolate the inclusion bodies. Next, the inclusion bodies were washed twice with 0.1× BugBuster, pelleted by centrifugation, and frozen at −20°C for later use. Then they were denatured in 8 M guanidine-HCl at pH = 8.5 on a rotator for 50 min at room temperature. Following a 16,000×g spin for 5 min, the denatured protein was then bound to Ni-NTA Superflow resin (Qiagen) that had been equilibrated in denaturing buffer [100 mM sodium phosphate (pH 8.0), 10 mM tris, 6 M guanidine-HCl]. The resin was loaded into a column and, using an AKTA Explorer system (GE Healthcare), the denatured protein was refolded on the column using a linear gradient to refolding buffer [100 mM sodium phosphate (pH 8.0) and 10 mM tris] over 4.5 h at a flow rate of 0.75 mL/min. Next the protein was eluted with a linear gradient to elution buffer [100 mM sodium phosphate (pH 5.8), 10 mM tris, and 500 mM imidazole] at 2 mL/min over 45 min. The protein fractions were diluted 5–10-fold into dialysis buffer [10 mM phosphate (pH 5.8)], filtered with a 0.2 µm syringe filter, and dialyzed. The concentration of rPrP^c^ was determined by measuring absorbance at 280 nm and purity was ≥99%, as estimated by SDS-PAGE (see Results), immunoblotting (data not shown), and mass spectrometry (mass = 16,238 amu; data not shown) [31]–[33]. rPrP^c^ preparations were aliquotted and stored at 0.2–0.4 mg/mL and −80°C.

### Tissue homogenate preparation

Hamster, deer and sheep 10% (w/v) BH's were prepared as previously described [30] and stored at −80°C. For dilution analysis, BH's were serially diluted (5–10-fold) in 0.1% SDS in phosphate buffered saline (PBS) containing 130 mM NaCl with N2 media supplement (Gibco) as a source of carrier protein [31]–[33]. Two microliter aliquots of the BH dilutions were used to seed RT-QuIC reactions.

Normal hamster muscle and spleen tissue were isolated, snap frozen in liquid nitrogen, and stored at −80°C until later use. Five 3 mm glass beads were placed in a 2 mL screw cap tube. Next, 0.1 g tissue was combined with 900 µL ice cold PBS and homogenized in two 30 s sessions with the mini beadbeater (Biospec Products) set to the homogenize setting. Further homogenization was accomplished with a mini 1.5 mL tube plastic mortar and pestle. Following a 5 min 1000×g clarification spin, the supernatant was collected, aliquotted and frozen for later use.

### Nasal lavage sample preparation

Weanling, Syrian golden hamsters (Simonsen Laboratories, Gilroy, CA) were i.c.-inoculated as described previously [24]. Collection of nasal lavages was also performed as previously described [24] and samples were stored at −80°C until use. After thawing, 4 µL nasal lavage was combined with 4 µL 0.05% SDS in PBS with N2 and serially diluted 5–10-fold for RT-QuIC dilution analysis. The amount of HY TME infectivity was measured in nasal lavages using a hamster bioassay. Fifty microliters of each nasal lavage in PBS was i.c.-inoculated into six Syrian hamsters and the time to onset of clinical symptoms was recorded. HY TME titer was inversely proportional to the incubation period and was estimated using the incubation interval assay as previously reported [24].

### Cerebral spinal fluid sample preparation

CSF samples from hamsters i.c.-inoculated with 263K were collected at the clinical stage of disease as previously described (Atarashi 2007). After thawing, 2 µL CSF was combined with 2 µL 0.1% SDS/PBS/N2 and serially diluted 5-fold for RT-QuIC dilution analysis.

### RT-QuIC method

RT-QuIC buffer (RTQB) composition was as follows: 10 mM phosphate buffer (pH 7.4), 130–500 mM NaCl, 0.1 mg/mL rPrP^C^, 10 µM ThT, and 10 µM ethylenediaminetetraacetic acid tetrasodium salt (EDTA). RTQB (92,96, or 98 µl, depending upon the volume of the seed samples) was loaded into wells of a black 96-well plate with a clear bottom (Nunc) and seeded with a 2–8 µl seed sample for final reaction volume of 100 µL. All reactions contained equivalent final concentrations of SDS (0.002%).

The plates were sealed with a plate sealer film (Nalgene Nunc International, catalog #265301) and then incubated in BMG Polarstar or Fluostar plate reader at 42°C for 20–68 h with cycles of 1 min shake (700 rpm double orbital) and 1 min rest throughout the incubation. ThT fluorescence measurements (450+/− 10 nm excitation and 480+/− 10 nm emission; bottom read, 20 flashes per well, manual gain of 2000, and 20 µs integration time) were taken every 15 min. For SDS-PAGE analysis, the RT-QuIC products were incubated with or without 3.5 µg/mL proteinase K (PK) in the presence of 0.27% N-lauroylsarcosine sodium salt for 1 h at 37°C followed by the addition of sample buffer, a 1 min high speed vortex, and a 10 min boil prior to analysis by SDS-PAGE and Deep Purple protein staining (GE Amersham).

### RT-QuIC analyses

Hamster 263K-inoculated BHs and CSFs and HY TME BHs were analyzed in the RT-QuIC with 8 replicates for each dilution, while nasal lavage samples were tested with 4 replicates. This study used four stocks of 10% BH from hamsters clinically affected with 263K scrapie (2 stocks from 2007 [37], [38], 1 stock from 1983, and 1 from 1998) that had been previously analyzed by hamster bioassay. Each stock was serially diluted as stated above and used to seed RT-QuIC reactions. Three dilution analysis experiments were done on separate days for each stock. In each experiment, eight replicates were used for each BH dilution except for the first experiment for each stock in which normal BH-seeded samples were run in replicates of four. Also, at least two different batches of rPrP^c^ and RTQB were used for each 263K stock analysis.

Within an individual experiment, the average variance in baseline ThT fluorescence from all 12 negative control experiments (320 individual wells) seeded with normal BH (NBH) was 5.4%. The plate reader measures ThT fluorescence in relative fluorescent units (rfu) with saturation occurring at ∼260,000. After a lag phase, virtually all prion seeded reactions rise to saturating fluorescence values (see next paragraph). We chose to run our BH dilution analysis reactions for 45 h because little sensitivity was gained beyond this time span. Nasal lavage samples were analyzed at 20 h to achieve optimal discrimination at the dilutions chosen.

A Spearman-Kärber analysis analogous to a bioassay's lethal dose 50% (LD_50_) [39] was used to estimate a seeding dose or dilution at which 50% of the wells became ThT positive (SD_50_). For this purpose, each well was considered analogous to an inoculated animal in a bioassay. In rare cases, the fluorescence in individual wells rose above baseline but did not reach saturation at ∼260,000 rfu within the duration of the reaction. To incorporate these intermediate reactions into Spearman-Kärber determinations of a SD_50_ value, we arbitrarily designated positive reactions as those with fluorescent readings of >200% of the average negative control fluorescence, although in our experience, these borderline cases were so rare as to scarcely influence the final SD_50_ values. For Spearman-Kärber analysis a dilution series with at least one dilution giving 100% ThT positivity and at least one dilution giving 0% ThT positivity was chosen. The 50% end-point was calculated using the following formula: *x*_p = 1_+1/2*d* - *d*∑*p* (*x_p_*_ = 1_: highest log dilution giving all positive responses; *d* =  log dilution factor; *p* =  proportion positive at a given dose; ∑*p* =  the sum of values of *p* for *x_p_*_ = 1_ and all higher dilutions).

### Spectroscopy

For circular dichroism (CD) spectroscopy, hamster rPrP^C^ 90-231 was at 0.3 mg/mL in 10 mM sodium phosphate buffer (pH 5.8). CD measurements were performed at 20°C on an OLIS DSM 17 CD spectrophotometer (On-Line Instrument Systems, Inc.) with the following parameters: fixed bandwidth  = 2 nm and constant integration time of 5 seconds. A quartz cylindrical cell with a 1 mm pathlength was used. Data were collected from 262–185 nm with 1 datum/0.55 nm with constant nitrogen gas purge. The resulting spectra were acquired in a single scan, followed by subtraction of the sodium phosphate buffer baseline, and incorporation of a 13-point Savitzky-Golay smoothing filter.

Fourier transform infrared spectroscopy (FTIR) was performed using a Perkin-Elmer Spectrum 100 instrument equipped with a diamond attenuated total reflectance sample unit and an MCT detector. For analysis of rPrP^C^ 90–231, 2.5 µl of a freshly thawed ∼0.3 mg/mL solution prepared as described above for use in RT-QuIC was applied to the diamond. 263K-seeded rPrP^res^ generated in a ThT-free RT-QuIC reaction and 263K PrP^Sc^ purified from hamster brain tissue [40] were pelleted, washed in water, and applied to the diamond as thick slurries containing 10–40 µg protein. After application to the diamond, the samples were dried under a stream of nitrogen while collecting serial background-subtracted spectra, each comprised of 64 accumulations (4 cm^−1^ resolution; 1 cm/sec OPD velocity; strong apodization). The rapid loss of the initially dominant liquid water signal (∼1640 cm^−1^) was followed until the protein amide I and II bands predominated and no further changes due to the evaporation of water were observed (within a few minutes).

### Ethics statement

Rocky Mountain Laboratories is an Association for Assessment of Laboratory Animal Care (AALAC)-accredited facility, and all animal procedures were carried out in strict accordance with the recommendations in the Guide for the Care and Use of Laboratory Animals of the National Institutes of Health. The protocol was approved by the institution's Animal Use and Care Committee and the National Institutes of Health (Protocol Number: 2003-11.4). All surgery was performed under isoflurane anesthesia, and all efforts were made to minimize suffering.

## Results

### A real-time QuIC assay for prions

**Figure 1:**
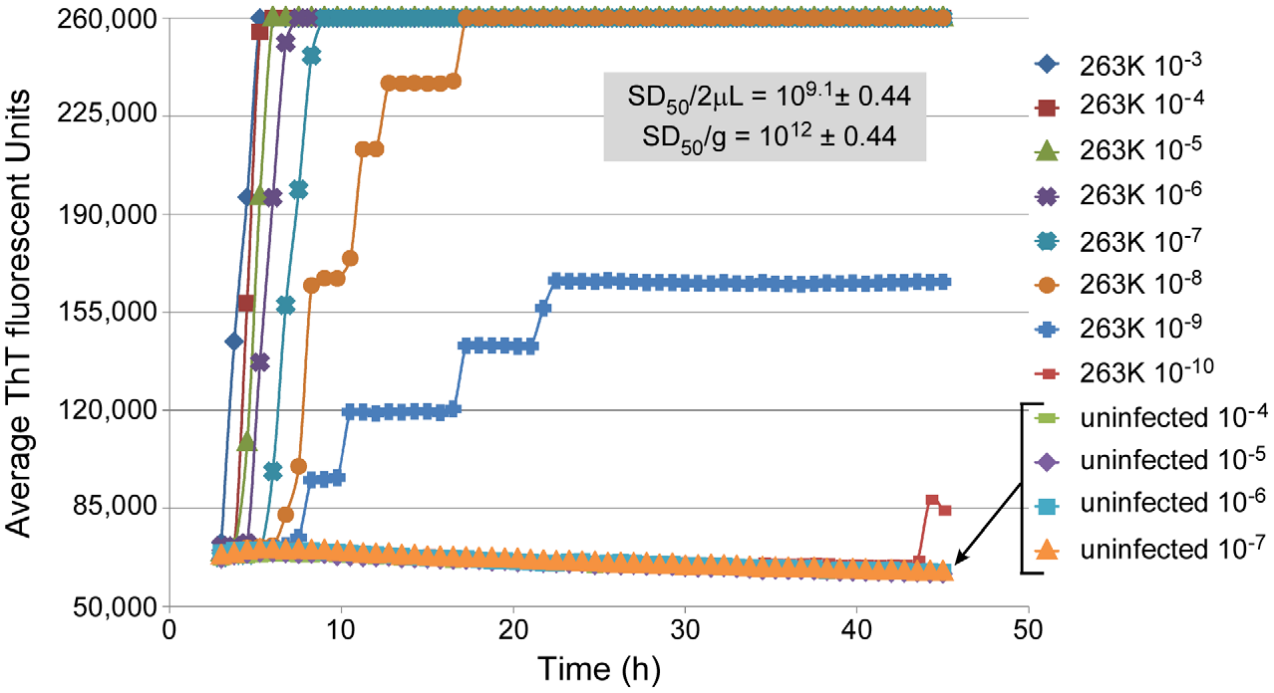
RT-QuIC sensitivity: analysis of dilutions of a scrapie brain homogenate stock. The designated dilutions of 263K scrapie or normal BH were used to seed RT-QuIC reactions containing recombinant hamster PrP^c^ 90-231 substrate. Each 0.75 h time point for each dilution is represented as an average ThT fluorescence from 8 replicate wells on a 96-well plate. The 50% seeding dose (SD_50_) is defined as the amount giving sufficiently enhanced ThT fluorescence in half of the replicate wells. In this case, the approximate SD_50_ was achieved with a 2 µl aliquot (the seed volume) of a 10^−9^ dilution of the scrapie BH stock. The Spearman-Kärber estimate of the SD_50_/2 µl neat brain tissue is shown, along with that adjusted to SD_50_/gram brain tissue.

The RT-QuIC assay incorporates rPrP^c^ as a substrate, intermittent shaking of the reactions in 96-well plates, detergent- and chaotrope-free reaction conditions, and ThT-based fluorescence detection of prion-seeded rPrP^c^ amyloid fibrils. As described for the ASA [34] and other ThT-based seeded amyloid fibrillizations assays [41], the advantage of using ThT detection is that it can be included in the reaction mixture. In the presence of amyloid fibrils, ThT undergoes an enhancement of fluorescence yield as well as a spectral shift in excitation/emission maxima that can be measured frequently over the RT-QuIC time course. In Figure 1, RT-QuIC reactions containing hamster rPrP^c^ 90–231 (comprised of residues 90–231) showed increased ThT fluorescence after lag phases of 2–4 h when seeded with a 10^−3^ dilution of BH from hamsters clinically affected with 263K scrapie. Each data point on the graph represents the average fluorescence from 8 replicate wells. The duration of the lag phase increased with higher dilutions of 263K brain. At 10^−9^ and 10^−10^ dilutions only 4 of 8 and 1 of 8 replicate wells, respectively, showed positive reactions. The rapid negative-to-positive conversion of individual wells often caused the stepwise increases in the average fluorescence from all wells. For dilutions of normal uninfected hamster brain (n = 32), no increase in ThT fluorescence was observed in this RT-QuIC assay. Because there was no gain in sensitivity for 263K-seeded samples and no increase in fluorescence in NBH control-seeded samples past 45 h (out to 75 h, not shown), we terminated our 263K experiments at 45 h. Developmental experiments that compared some conditions of the ASA and RT-QuIC reactions indicated that the retardation of spontaneous amyloid formation in the latter was largely due to the lack of guanidine hydrochloride (Fig. S1), the choice of appropriate NaCl concentrations (see below), and the use of intermittent, rather than continuous, shaking (data not shown).

The observation that diminishing proportions of the replicate RT-QuIC reactions were positive at extreme dilutions of the scrapie BH seed (Fig. 1) was reminiscent of TSE animal bioassays [27]. In the latter, the Spearman-Kärber [39] or Reed-Muench [42] methods are commonly used to estimate infectivity titers in the original samples [e.g. 50% lethal dose (LD_50_) per mL] based on the proportion of diseased animals at each dilution. By analogy, we applied the Spearman-Kärber method to prion seeding activities by determining the dilution, or seeding dose (SD), giving positive RT-QuIC responses in 50% of replicate reactions (SD_50_). The SD_50_ concentrations in the undiluted samples were then calculated to provide a quantitative comparison of relative prion seeding activities. For example, analysis of the data shown in Figure 1 indicated that the original scrapie brain tissue had 6×10^11^ SD_50_ per g (Table 1, Brain #4).

**Table 1:** RT-QuIC and animal bioassay end point dilution analyses.

263K Stock	RT-QuIC (log SD_50_ per g brain*)				Bioassay
	1^st^ Rxn	2^nd^ Rxn	3^rd^ Rxn	Avg ± Std Dev	log LD_50_ per g Brain*
Brain #1	12.1	12.0	11.6	11.9±0.26	10.2
Brain #2	11.2	11.3	11.6	11.4±0.19	9.80
Brain #3	11.2	11.5	11.8	11.5±0.31	10.7
Brain #4	11.3	12.2	11.8	11.8±0.44	10.4

*calculated by Spearman-Kärber analysis as described in Materials and Methods.

### Characterization of RT-QuIC reaction products

**Figure 2:**
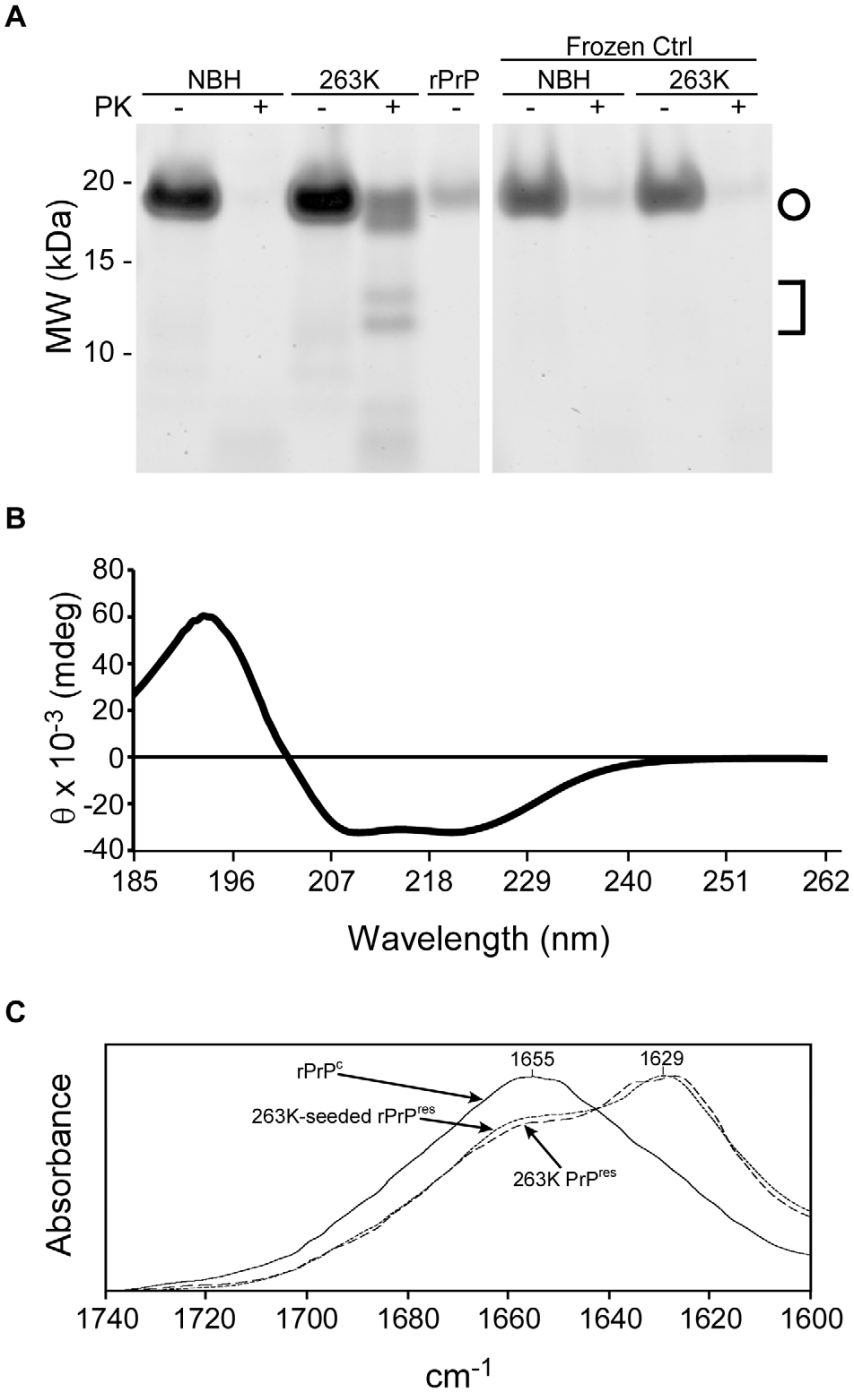
SDS-PAGE, circular dichroism (CD), and Fourier transform infrared spectroscopy analyses of RT-QuIC products. RT-QuIC reactions were seeded with 5×10^−6^ dilution of either NBH or 263K BH (containing 100 fg PrP^Sc^). (A) Products were PK digested and analyzed by SDS-PAGE. The gel was stained with a non-specific protein stain (Deep Purple). The circle indicates the ∼18 and 19 kDa bands while the bracket represents the ∼13 and 14 kDa bands in the PK-digested products of the scrapie-seeded reaction. (B) CD spectrum of the initial hamster rPrP^C^ 90-231 substrate for RT-QuIC reactions. (C) FTIR spectra of hamster rPrP^C^ 90-231, 263K-seeded rPrP 90-231 RT-QuIC product, and PK-treated brain-derived 263K PrP^Sc^.

To test whether the products of a ThT-positive reaction in the RT-QuIC assay are similar to those seen previously in our detergent based QuIC assay, PK-treated RT-QuIC products were analyzed by Deep Purple non-specific protein staining of SDS-PAGE gels. 263K scrapie brain-seeded reactions produced a distinct pattern of PK-resistant bands of ∼20, 18, 14 and 13 kDa, whereas those seeded with normal tissue and those put on ice without shaking and incubation cycles produced virtually no PK-resistant polypeptide products (Fig. 2A). The 18-, 14- and 13-kDa polypeptide bands were similar to those observed in the scrapie-seeded products of the original QuIC reactions [32] which were performed using full-length rPrP^c^ 23-231, rather than rPrP^c^ 90-231, as a substrate. The 20-kDa polypeptide band appeared to be a nearly full length form of the rPrP^c^ 90-231 substrate, which was not observed in the original QuIC reactions using the rPrP^c^ 23-231 substrate [32]. Quantitative comparison of band intensities of 263K scrapie-seeded RT-QuIC products with and without PK treatment indicated that the PK-resistant products accounted for 50–60% of the input rPrP^c^ substrate (data not shown). The 263K PrP^Sc^ content of the seed for this reaction was ∼10,000-fold lower than would be detectable by Deep Purple stain and thus was not visible (data not shown).

We also assessed the conformational changes in the rPrP^C^ 90-231 substrate induced by prion seeding in the RT-QuIC by spectroscopic methods. The circular dichroism (CD) spectrum of the initial hamster rPrP^C^ 90-231 substrate showed a minimum at 208 nm and a shoulder at 222 nm (Fig. 2B) that is indicative of alpha helix content and similar to that reported for native PrP^C^ [43]. The Fourier transform infrared (FTIR) absorbance maximum at 1655 cm^−1^ (Fig. 2C) was also consistent with high alpha helix content and similar to previous spectra of rPrP^C^ in water [44], [45]. In contrast, the product of 263K prion-seeded RT-QuIC reactions had an FTIR absorbance maximum at 1627 cm^−1^, which is indicative of a high beta sheet content similar to that of brain-derived, PK-treated 263K PrP^res^ (Fig. 2C) [46]. Thus, the prion-induced secondary structure changes in rPrP^C^ 90-231 were at least grossly similar to those occurring naturally upon conversion of PrP^C^ to PrP^Sc^ [43], [47].

### RT-QuIC reproducibility and comparison to bioassay

**Figure 3:**
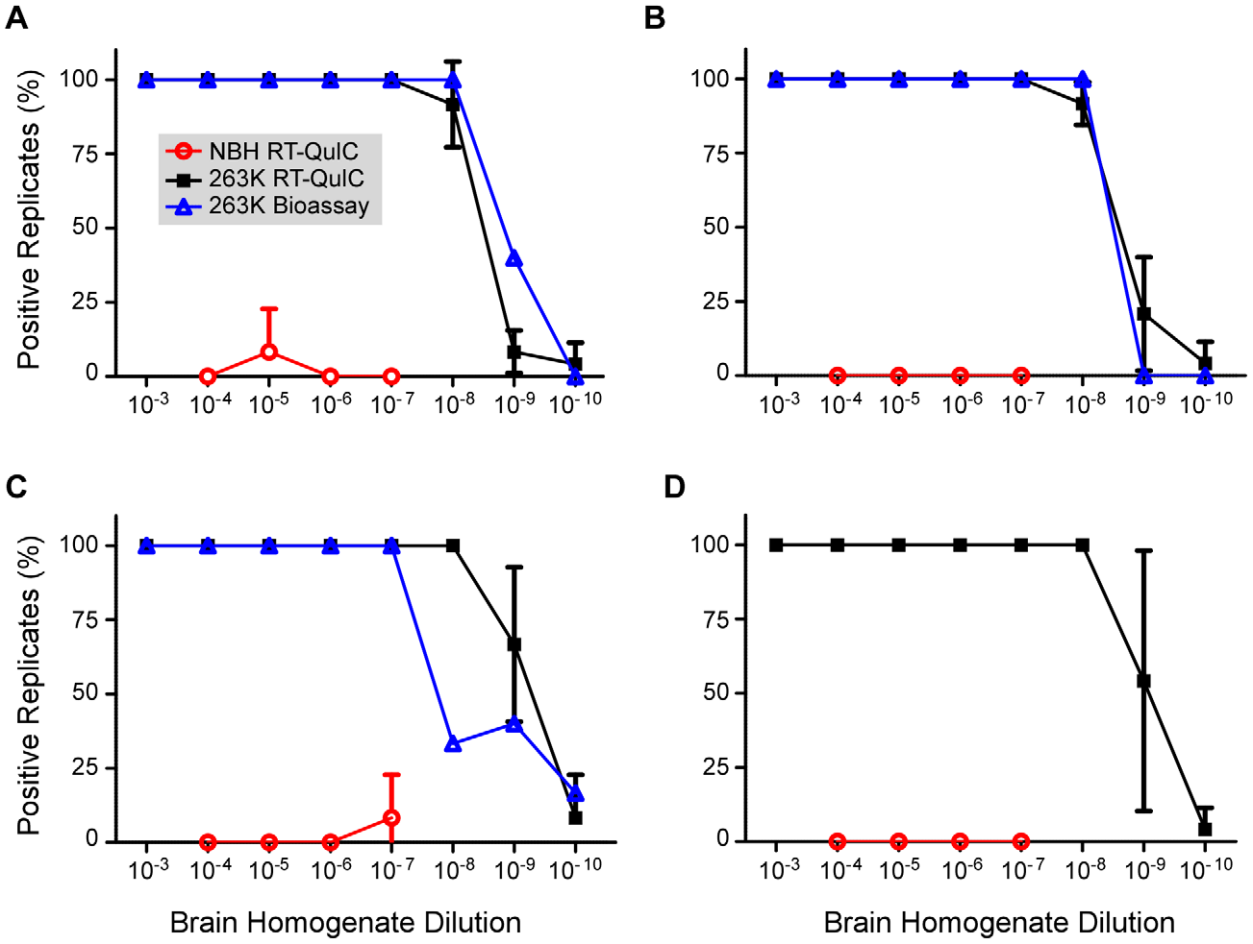
Combined RT-QuIC end-point dilution analyses from four scrapie BH stocks. Each 263K stock (Brain #1-#4 corresponding to Panels A-D) was serially diluted and used to seed RT-QuIC reactions. The percentage of replicate wells (n = 4–8) that are ThT-positive according to the criteria in Material and Methods was calculated and the mean percentage ± standard deviation from three separate experiments with each stock is shown. Animal bioassay data is represented as the percentage of replicate animals reaching the clinical stage of disease at each dilution of 263K scrapie BH (4–22 animals per dilution per stock).

To assess the reproducibility of the RT-QuIC assay and to compare its sensitivity to that of infectivity bioassays, we performed end point dilution analyses of normal and clinical 263K scrapie brain homogenate stocks (four independent stocks of each), the latter having been titered by end-point dilution bioassay in hamsters. Ten-fold serial dilutions of each stock were subjected to three separate RT-QuIC analyses. In the first analysis for each stock, only 4 replicates per dilution of NBH were used, however 8 replicates were used for all other dilutions and analyses. At least 2 independent batches of buffers and hamster rPrP^c^ 90-231 substrate were used for the three analyses of each stock. Figure 3 shows the percentage of RT-QuIC-positive wells occurring within 45 h as a function of dilution for each pair of simultaneously assayed normal and 263K hamster scrapie brain stocks. For each stock, 100% of the replicate reactions were positive down to 10^−7^ dilution, but more dilute samples (10^−8^-10^−10^) gave lower percentages of positive replicates. In these experiments, two out of the 240 negative control wells seeded with normal BH gave positive responses as shown by the non-zero NBH values in Figure 3A and C. Whether these apparent false positive reactions were due to rare spontaneous fibril formation or to inadvertent prion contamination of the individual wells is unclear. For comparison, the percentage of replicate animals that were positive in the hamster bioassays of the three scrapie BH stocks is also shown (Fig. 3A, B, and C). The data illustrate how, in both the bioassay and RT-QuIC, the proportion of positive replicates decreased at similar dilutions of the 263K BH. Table 1 summarizes the results of Spearman-Kärber calculations of the SD_50_ and LD_50_ per gram brain determined by RT-QuIC and hamster bioassay, respectively. Although the data in Figure 3 showed similar end-point dilutions for the two types of assays, the calculated SD_50_/g values were 0.35–1.5 log higher than the LD_50_/g values. These differences can largely be attributed to the fact that 25-fold smaller sample volumes were tested in the RT-QuIC assays. Overall, the data show that the RT-QuIC and bioassay are roughly comparable in sensitivity.

**Figure 4:**
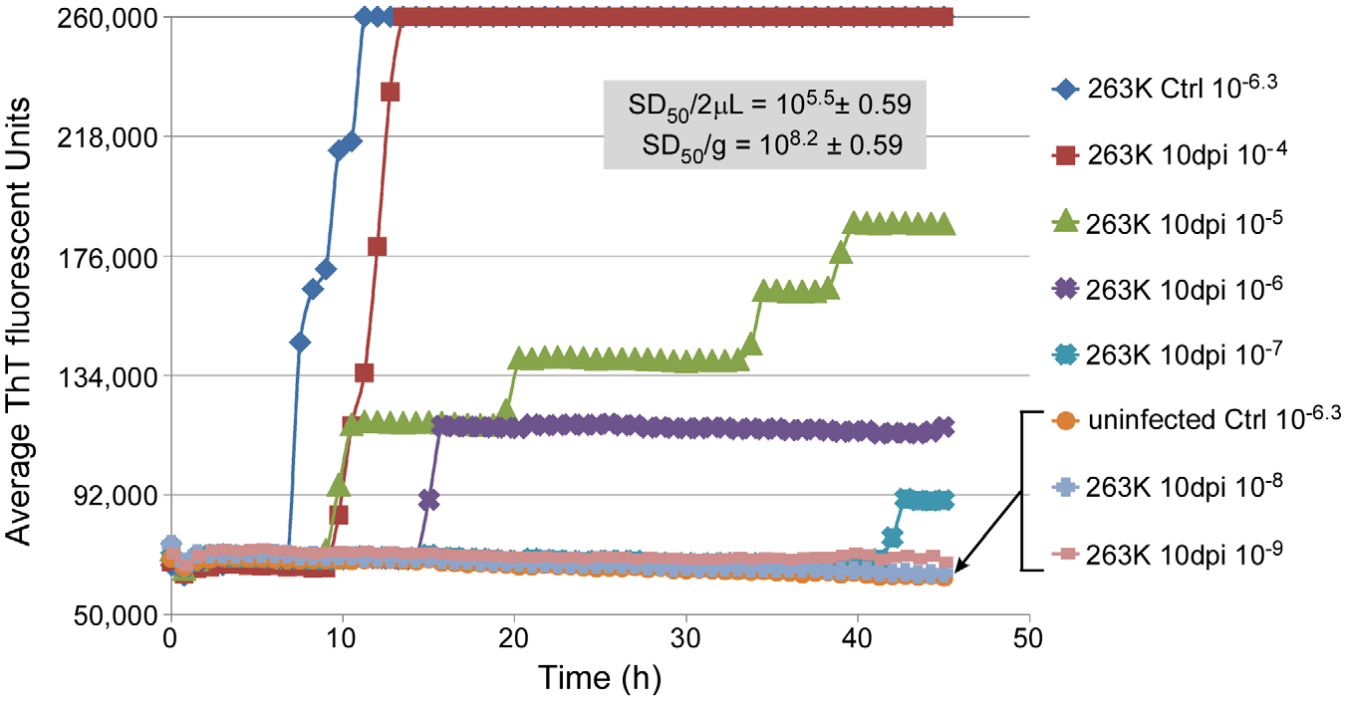
RT-QuIC end-point dilution analysis of three 263K-inoculated preclinical 10 dpi hamster BHs. Hamsters were i.c.-inoculated with 263K scrapie BH. Animals were sacrificed at 10 dpi and brains were analyzed with RT-QuIC dilution analysis. Eight replicate wells were used for each BH dilution. The average Spearman-Kärber estimates of the SD_50_/2 µl and SD_50_/g neat brain tissue from three animals are shown.

The above analyses were performed on brain tissue harvested from clinically affected hamsters at 80–85 dpi after inoculation with 50 µl of a 1% 263K scrapie BH. To determine if brain SD_50_ concentrations increased with time after i.c. scrapie inoculation we performed end-point dilution RT-QuIC on brains harvested at 10 dpi and calculated a mean SD_50_ concentration of 10^8.2^/g brain (Fig. 4). The ∼4 log_10_ increase in SD_50_/g between 10 dpi and the clinical phase (Fig. 4 and 1) was comparable to the estimated 3-4 log_10_ increase in infectivity (LD_50_/g) that was reported in a similar timeframe in hamsters inoculated with slightly higher i.c. doses of 263K BH [48]. Thus brain RT-QuIC SD_50_ concentrations increased with the expected rise in infectivity levels after inoculation.

### RT-QuIC analyses of nasal lavages

**Figure 5:**
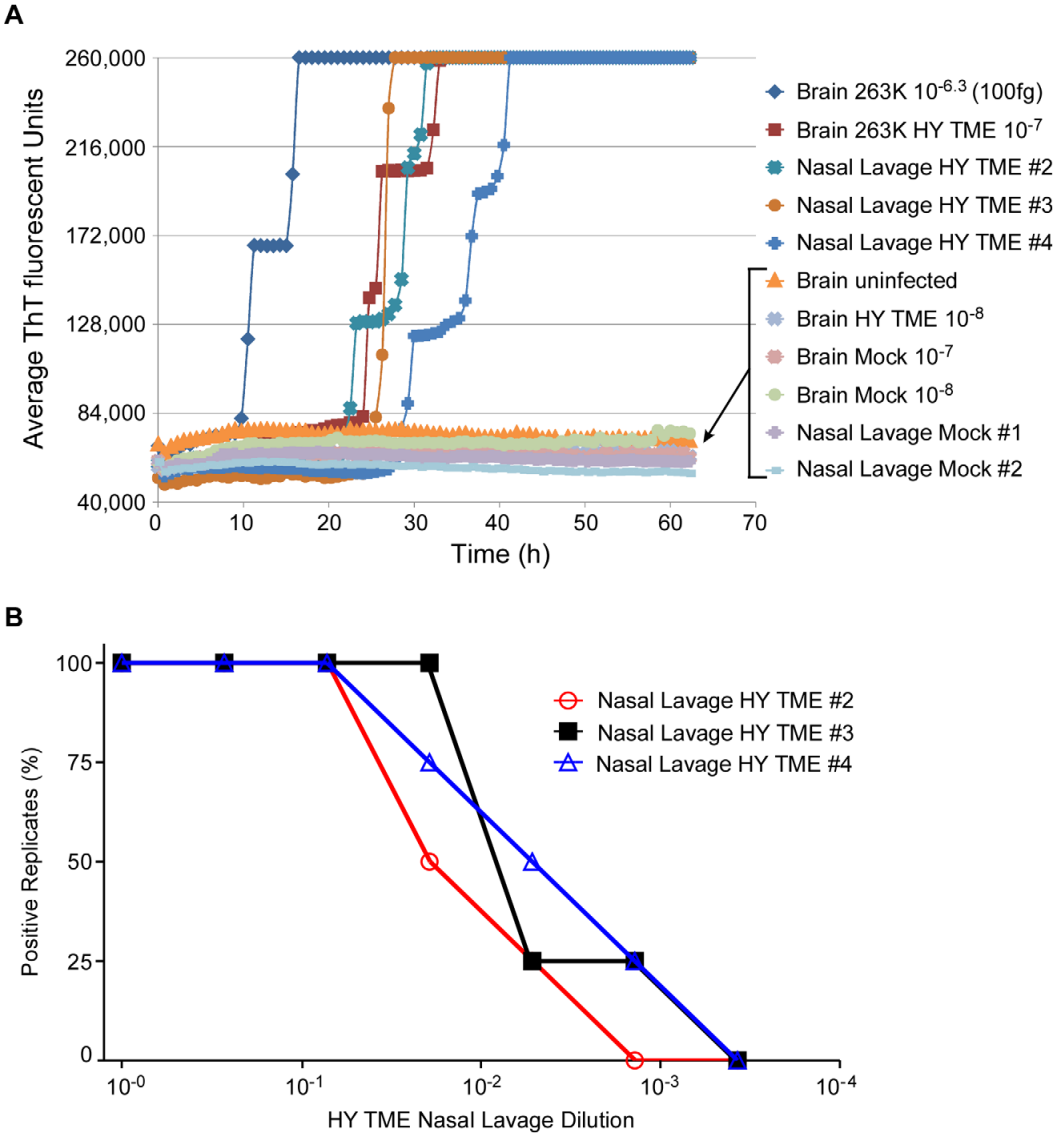
RT-QuIC detection and end point dilution analysis of nasal lavages from HY-TME-infected hamsters. At the clinical stage of disease in hamsters i.c.-inoculated with HY-TME, nasal lavage samples (∼1 ml) were collected from individual hamsters, serially diluted and used to seed RT-QuIC reactions. (A) Detection of HY-TME in nasal lavages is shown. As positive and negative controls, 5×10^-6^-fold dilutions of hamster scrapie BH (containing ∼100 fg PrP^Sc^) and NBH, respectively, were used as seeds. Dilutions of HY-TME brain were also tested for comparison. The data points show the average ThT fluorescence of 8 replicate wells. (B) Three representative HY-TME infected hamster lavages were serially diluted and analyzed by RT-QuIC. Each data point represents the percentage of replicate wells (n = 4) that are ThT-positive according to the criteria in Material and Methods as a function of nasal lavage dilution factor. The tabular data from lavages tested here can be found in Table 2.

Recently prions have been detected in nasal lavages from hamsters clinically affected with the Hyper (HY) strain of hamster-adapted transmissible mink encephalopathy (TME) by hamster inoculation and the original immunoblot-based QuIC reaction [24]. To quantitate the amount of prion seeding activity in lavages and to evaluate the RT-QuIC assay with another prion strain and tissue source, we analyzed coded samples of nasal lavages from HY-TME-infected and normal hamsters, as well as lavage buffers spiked with dilutions of HY-TME BH. As shown in Figure 5A, 2 µL aliquots of lavage buffer spiked with a 10^−7^ dilution of HY-TME brain tissue were positive in all replicate RT-QuIC reactions whereas further 10-fold dilutions were negative. All unspiked neat nasal lavage samples from HY-TME-infected animals were positive and by comparison, all negative control nasal lavages from normal hamsters, and all lavage buffer samples spiked with normal BH were negative in replicate reactions (Fig. 5). These results showed that prions were readily detected in nasal lavages from HY-affected hamsters by RT-QuIC.

We then performed end point dilution analyses of eight nasal lavage samples from HY-TME infected hamsters. Data from three representative nasal lavage analyses is shown in Figure 5B. The calculated SD_50_ concentrations for all the lavages are shown in Table 2 and compared to incubation times and attack rates from intracerebral inoculations of neat 50 µL aliquots of those lavages into hamsters. The hamster inoculations indicated the presence of prion infectivity in the lavages. However, the extended and variable incubation periods make it difficult to estimate relative infectivity levels in most of these samples based on the hamster inoculations without bioassay end point dilution analyses. Quantification by the RT-QuIC gave SD_50_ concentrations that were as high as 10^5.7^ per mL lavage. Considering the major dilution effect of rinsing the nasal cavity of hamsters with 1 mL of buffer, these results indicate that substantial concentrations of infectivity can be present in endogenous nasal fluids of HY TME-infected hamsters.

**Table 2:** HY-TME nasal lavage analyses: comparison of RT-QuIC SD_50_ with incubation period in hamsters.

Nasal Lavage (Animal number)	RT-QuIC	Inoculations	
	log SD_50_/mL*	Avg Incubation Time (d) ± Std Dev	Clinical/Inoculated
#1	4.7	114±13.1	6/6
#2	4.7	118±9.50	6/6
#3	5.2	121±13.8	6/6
#4	5.7	125±11.3	6/6
#5	4.2	151±48.1	5/6
#6	4.2	185±44.0	6/6
#7	4.2	186±27.5	5/6
#8	3.5	192±37.0	3/6

*calculated by Spearman-Kärber analysis as described in Materials and Methods.

### Prion seeding activity in CSF

**Figure 6:**
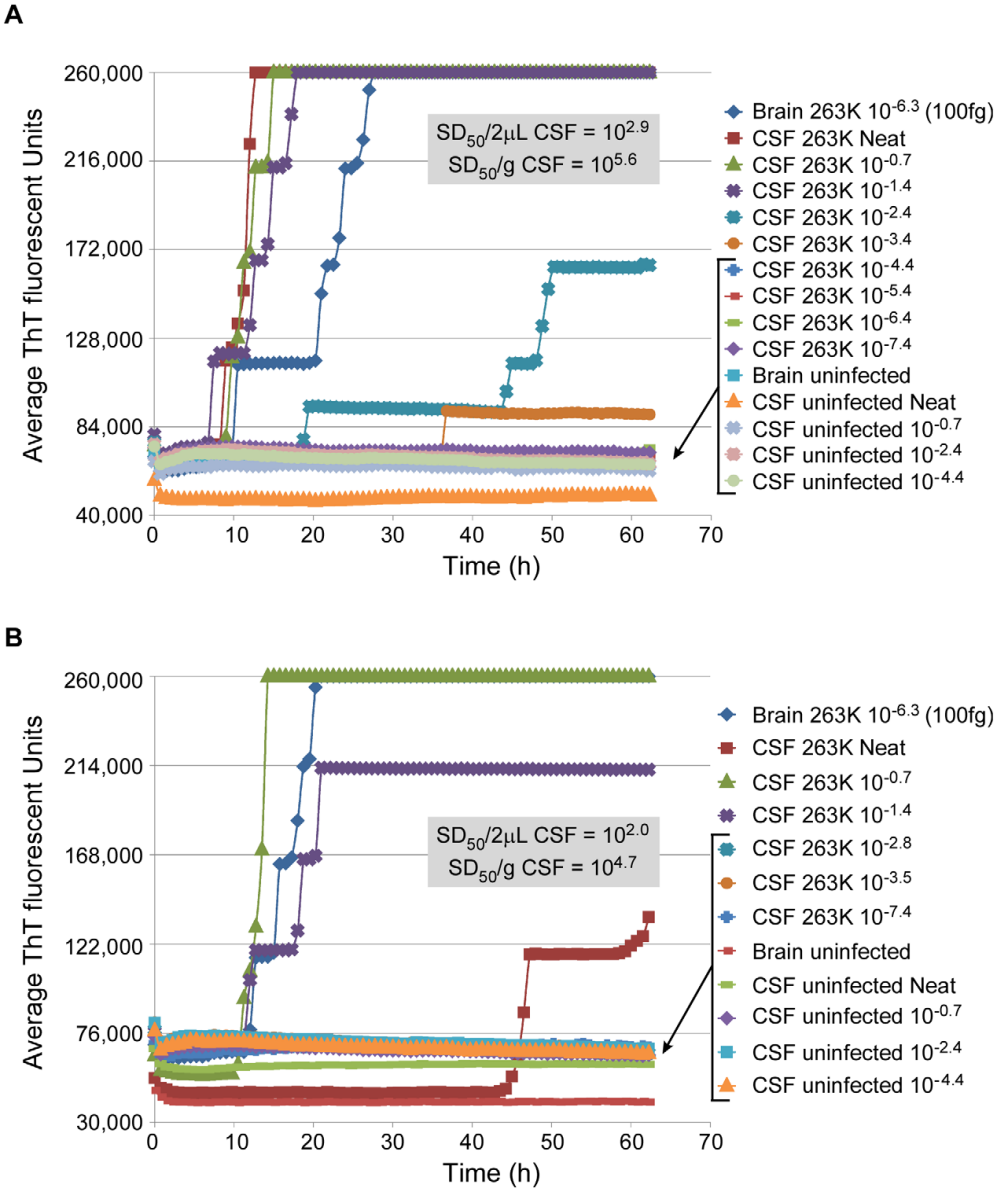
RT-QuIC end-point dilution analysis of CSF from scrapie-infected hamsters. CSF was collected at the clinical stage of disease from two individual hamsters (Panel A and B) i.c.-inoculated with 263K scrapie BH. CSF was serially diluted 5-fold and analyzed by RT-QuIC. Each 0.75 h time point for each dilution is represented as an average ThT fluorescence from 8 replicate wells. The average Spearman-Kärber estimates of the SD_50_/2 µl (the seed volume) and SD_50_/ml neat CSF are shown.

In order to quantitate the prion seeding activity in CSF, we performed endpoint dilution analysis on CSF samples from two hamsters that were clinically affected with 263K scrapie. Values of 10^5.6^ and 10^4.7^ SD_50_ per mL were obtained (Fig. 6). One of the CSF samples (Fig. 6B) was tinted slightly red, presumably from contamination by blood, whereas the other CSF sample (Fig. 6A) was clear. In the former case, the RT-QuIC reactions seeded by the neat CSF were inhibited relative to those seeded with the 10^−0.7^ and 10^−1.4^ dilutions. We suspect that, consistent with our failed attempts to detect prions directly in blood or scrapie-spiked blood (data not shown), blood contaminants in the neat red-tinted CSF sample inhibited the RT-QuIC reactions, but were rapidly diluted to subinhibitory levels.

### RT-QuIC tolerances for tissue components

**Figure 7:**
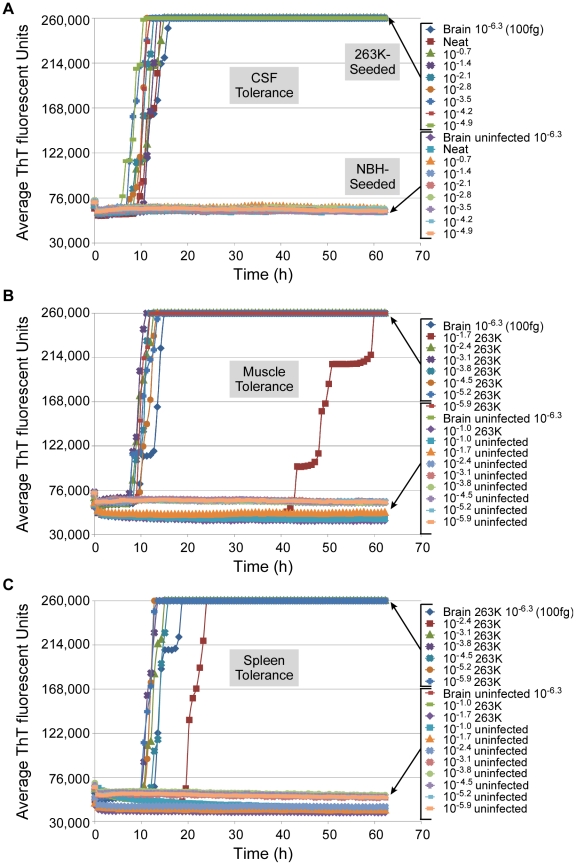
Tissue tolerance of RT-QuIC assay. RT-QuIC reactions with recombinant hamster 90-231 PrP^c^ substrate were seeded with 2 µL neat or diluted samples of cerebral spinal fluid (A), 10% muscle (B), or 10% spleen (C), each spiked with a 10^−6.3^ dilution of normal or 263K scrapie-infected (clinical) hamster BH. Each 0.75 h time point for each dilution is represented as an average ThT fluorescence from 4 replicate wells.

To explicitly test for interference of the RT-QuIC by components of CSF and other tissues, we spiked neat CSF or 10% homogenates of spleen and muscle from normal hamsters, or serial 5-fold dilutions thereof, with a 10^−6.3^ dilution (100 fg PrP^Sc^) of hamster 263K scrapie BH or normal BH. We observed partial-to-complete inhibition of the RT-QuIC reactions seeded with 2 µl seed aliquots containing 2–10% (10^−1.7^-10^−1.0^) muscle tissue and 0.4–10% (10^−2.4^-10^−1.0^) spleen tissue, but no inhibition by clear CSF (Fig. 7). However, further dilutions of the muscle and spleen tissue relieved the inhibition. In other experiments, we also have observed inhibition by ≥1% hamster brain tissue and 100% human plasma (data not shown). Thus, concentrated tissue components from various tissues (e.g. muscle, spleen, brain and blood) can interfere with the RT-QuIC reaction, making it important to reduce those components to subinhibitory concentrations.

### Adaptation of RT-QuIC to other species

**Figure 8:**
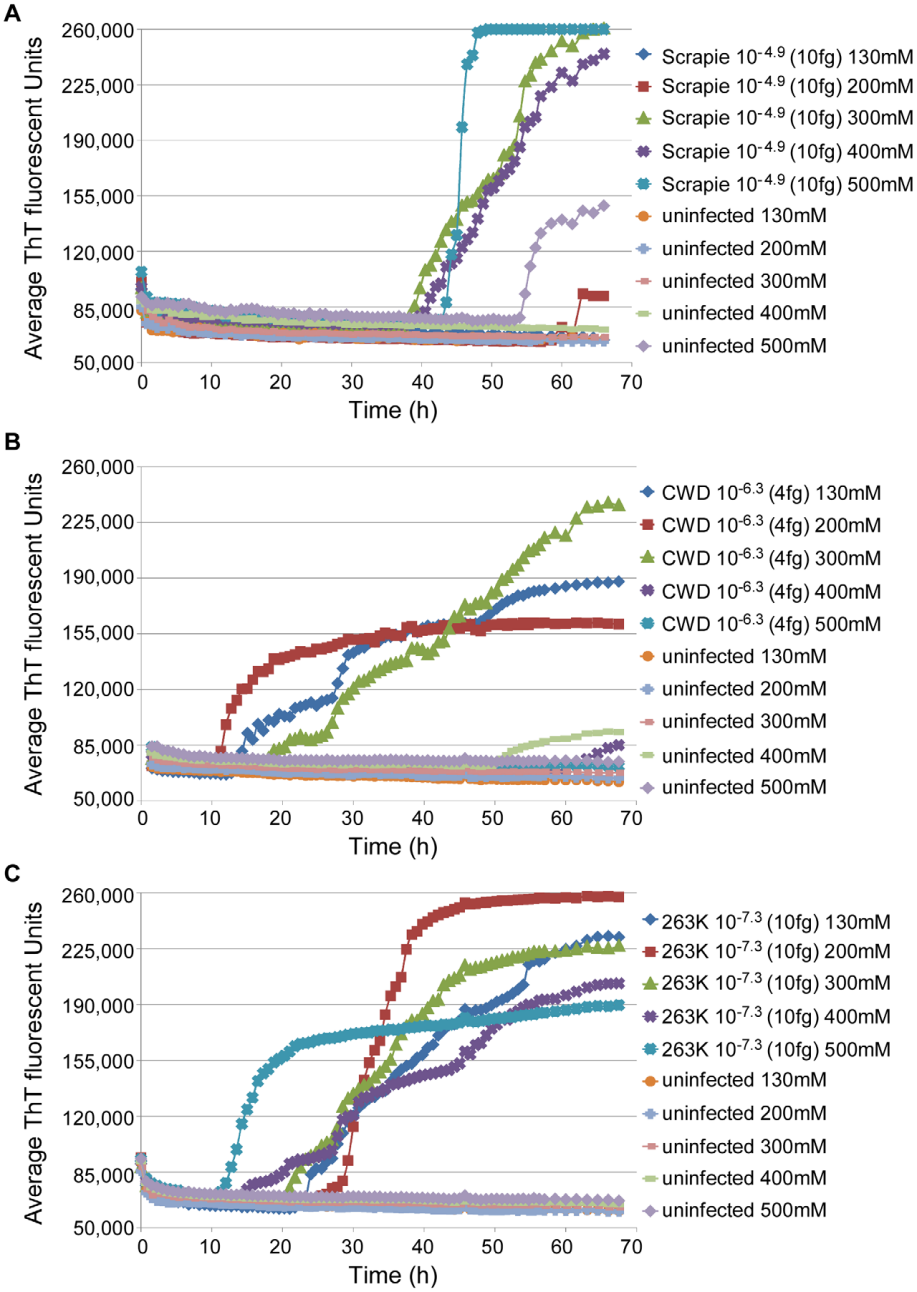
Sodium chloride titration for sheep, deer, and hamster RT-QuIC reactions. Dilutions of sheep scrapie (10^−4.9^; ∼10 fg PrP^Sc^) (A), deer CWD (10^−6.3^; ∼4 fg PrP^CWD^) (B), and hamster scrapie (10^−7.3^; ∼10 fg PrP^Sc^) (C) BH's and corresponding dilutions of NBH of the same species were used to seed RT-QuIC reactions. The NaCl concentration in the reactions was varied as designated. All reactions utilized the homologous full length rPrP^C^ substrates. The data points show the average ThT fluorescence of 4–8 replicate wells.

To test the applicability of the RT-QuIC assay to other prion disease susceptible species, we seeded RT-QuIC reactions with BH's from chronic wasting disease (CWD)-infected deer and scrapie-infected sheep, as well as negative control animals. In these reactions, full-length cervid (PrP genotype G_96_M_132_S_138_S_225_Q_226_), sheep (PrP genotype V_136_R_154_Q_171_), and hamster rPrP^c^ substrates homologous with the PrP^Sc^ molecules in the seed materials were used, except in the case of the reactions seeded with uninfected negative control sheep brain homogenate. In this case, we lacked the matching negative control brain material (i.e., VRQ) and had to substitute brain homogenate from a slightly different scrapie-susceptible sheep genotype (A_136_R_154_Q_171_). When reaction conditions like those of the hamster scrapie-compatible reaction described above were used with concentrated seeds, e.g. 10^−4^ dilutions of infected brain tissue, all replicate reactions were positive within 30 h and 40 h for the CWD and sheep scrapie seeds, respectively (data not shown). To further optimize these assays for detecting low levels of prions, we varied the NaCl concentrations in reactions seeded with highly diluted seed samples (10^−6^-10^−7^; 4-10 fg PrP^Sc^ equivalents). Good discrimination between prion-seeded and negative control reactions was found with 300–400 mM NaCl for the sheep reactions (Fig. 8A) and 200–300 mM NaCl for the deer reactions (Fig. 8B). The latter conditions were also optimal for 263K scrapie-seeded RT-QuIC reactions using full-length hamster rPrP^c^ substrate (Fig. 8C). At these respective NaCl concentrations, negative control reactions from all species were negative (n = 76) for 68 h. Higher NaCl concentrations tended to reduce the prion-seeded positive reactions and/or enhance the likelihood of apparently spontaneous positive responses in negative control reactions. In summary, these results show that the RT-QuIC can be applied to the detection of prions from deer and sheep brain infected with CWD and scrapie, respectively.

## Discussion

The RT-QuIC is a rapid prion detection assay that is more amenable to high-throughput applications than the original QuIC and much less prone to generate spontaneous, unseeded positive reactions than the ASA assay. The sensitivity of the RT-QuIC is similar to the *in vivo* bioassay in hamsters, but is roughly 50–200 times faster and much less expensive.

Using this assay, we have been able to rapidly detect and quantify prion seeding activities in nasal lavages from clinically TME-affected hamsters. Considering that nasal lavages are likely to dilute endogenous nasal cavity fluids by at least 100-fold, these results confirm and extend a previous report of substantial prion infectivity in nasal secretions from hamsters in the clinical phase of HY TME infection [24]. In the previous report, we detected nasal fluid prions by bioassay and the original immunoblot-based QuIC assay. In the current study, our ability to rapidly detect and quantitate prion seeding activity in nasal lavages using the RT-QuIC raises the possibility that such testing of nasal lavages or swabs could help in diagnosing prion disease infections of humans and animals on a high-throughput basis.

Our detection and quantitation of prion seeding activity in the CSF of 263K scrapie-infected hamsters suggests that CSF, being a relatively accessible specimen, should be collected for prion disease diagnosis by RT-QuIC. Interestingly, the CSF SD_50_ levels (10^5.7^ and 10^4.6^/ml) (Fig. 8) were similar to the highest value obtained for nasal lavages (10^5.7^/ml) (Table 2). However, the CSF should have at least 100-fold lower levels of prion seeding activity than the endogenous nasal fluids, given the considerable dilution that occurs when the nasal cavities are flushed with lavage buffer.

The origin of the rare positives that we observed in negative control RT-QuIC reactions (Fig. 3) is difficult to ascertain. Because we simultaneously tested both positive and negative controls on the same plates, there was some, but obviously very low, potential for prion seeds to be inadvertently transferred from prion-seeded wells to adjacent negative control reactions. Moreover, given the very high sensitivity of the assay, even a minute contamination could elicit a false-positive reaction. Yet another explanation could be a cross contamination due to a failure of our plate sealer tape during the course of the reaction incubation. Fortunately, whether due to contamination or spontaneous amyloidogenesis, such apparent false positives are extremely rare and can simply be retested for confirmation.

It is likely that the same multimeric particles of abnormal PrP that stimulate conversion of PrP^C^ or rPrP^c^ to an abnormally folded form in *in vitro* reactions also cause prion “infections” *in vivo*. Consistent with this idea, we found that positive RT-QuIC reactions were obtained only with seeds derived from TSE-infected animals (except for the rare exceptions described above). Moreover, we obtained similar end-point dilutions of scrapie BH with both the bioassay and the RT-QuIC (Fig. 3). These results gave the appearance of a direct quantitative correspondence between the activities measured in these assays. Indeed, we expect that for prions of a particular strain and tissue source, there will be a proportional relationship between the activities measured by end-point dilution analyses with the RT-QuIC and animal bioassay. However, the sensitivities of these distinct assays will likely be influenced by some fundamentally different factors *in vitro* and *in vivo* and should not be expected to coincide as closely as they have in Figure 3 with all types of prion samples or all permutations of the assays. Indeed, further studies will be required to determine whether RT-QuIC assays detect naturally occurring PrP aggregates that are associated with familial PrP mutations and disease, but are non-infectious in bioassays. This anticipated variability of the RT-QuIC and bioassay with different prion sample types does not diminish the utility of the RT-QuIC in assessing the relative amount of prion seeding activity in samples of similar nature. In further developments of RT-QuIC assays for certain purposes, e.g. diagnostic testing, the possibility that certain abnormal non-PrP amyloids could give false positive RT-QuIC reactions should also be considered.

The end-point dilution strategy for determining relative seed concentrations should be applicable to amyloid seeding assays for a variety of misfolded protein aggregates regardless of the means of detecting the amyloid product, e.g. by ThT fluorescence as in the ASA [34] and RT-QuIC assays, or immunoblotting as in PMCA [8], rPrP-PMCA [31] or original QuIC [32] assays. Like the RT-QuIC, many amyloid-seeded polymerization reactions progress rapidly to completion after a lag phase, providing an all-or-nothing response within appropriately selected time frames. This typical feature of seeded polymerization reactions should facilitate determinations of the proportion of positive reactions among replicates at a given sample dilution. Analyses of data from serial dilutions of various samples using the Spearman-Kärber [39] or Reed-Muench [42] algorithms can improve estimates of SD_50_ values per unit volume, which then indicate the relative concentrations of seeding activity in the samples.

As noted above, Chen and colleagues have recently described an alternative means of obtaining quantitative estimates of prion seeding activity using PMCA reactions, called qPMCA [36]. Rather than assaying serial dilutions of a sample and determining the end point dilution, as we demonstrate here, a single sample dilution is assayed in serial PMCA reactions and the relative seeding activity is estimated from the number of serial PMCA rounds that are required to detect a positive response. The accuracy of qPMCA therefore depends on the strength of the inverse correlation between the prion seed concentration and number of rounds required. Although these investigators have documented such a correlation, its biochemical/kinetic basis remains unclear. In contrast, end-point dilution analyses can simply be explained as a titration of the active species to the detection limit. Further studies will be required to determine which approach to estimating relative prion concentrations is more robust and practical for comparing specific sample types.

Within individual RT-QuIC experiments composed of multiple, simultaneous reactions, we observed a clear dependence of the lag phase on the concentration of seed, as illustrated in Figure 1. The lag phase might be considered analogous to the TSE incubation period between the inoculation and the near terminal stage of disease. In certain combinations of host and TSE strain, standard curves correlating bioassay incubation period with inoculated dose can be established and used to determine relative prion infectivity levels in unknown samples without resorting to more time-consuming and animal-intensive end-point dilution analyses. An analogous correlation between prion seed concentration and lag phase in the seeding assays like the RT-QuIC or ASA might also allow for seeding activity estimation without testing serial dilutions of each unknown. However, further work will be required to determine the efficacy, reproducibility, and validity of such an approach. In the mean time, the end-point dilution approach described in the current manuscript provides a clear means of quantitating prion seeding activity.

In summary, the end-point dilution RT-QuIC analysis provides quantitative comparisons of prion seeding activity. Although the extent to which prion seeding activity correlates quantitatively with infectivity *in vivo* under various other circumstances remains to be determined, we have shown that the RT-QuIC assay provides rapid and highly sensitive discrimination of prion-infected and uninfected brain tissues, nasal lavages, and CSF.

## Supporting Information

Figure S1 Guanidine HCl effects in RT-QuIC reactions seeded with sheep scrapie and deer CWD. Dilutions of sheep scrapie (10^-3.9^; ∼100 fg PrP^Sc^) (A) and deer CWD (10^-5.3^; ∼40 fg PrP^CWD^) (B) BH's and corresponding dilutions of NBH from the same species were used to seed RT-QuIC reactions. The guanidine-HCl concentration in the reactions was varied as designated. All reactions utilized the homologous full length rPrP^C^ substrates. The data points show the average ThT fluorescence of 4 and 8 replicate wells respectively.
(0.24 MB TIF)
