# Supplementary figures and images for: Rapid End-Point Quantitation of Prion Seeding Activity with Sensitivity Comparable to Bioassays
Source: PLoS Pathog. 2010 Dec 2;6(12):e1001217. doi: 10.1371/journal.ppat.1001217 (PMC2996325; doi:10.1371/journal.ppat.1001217)

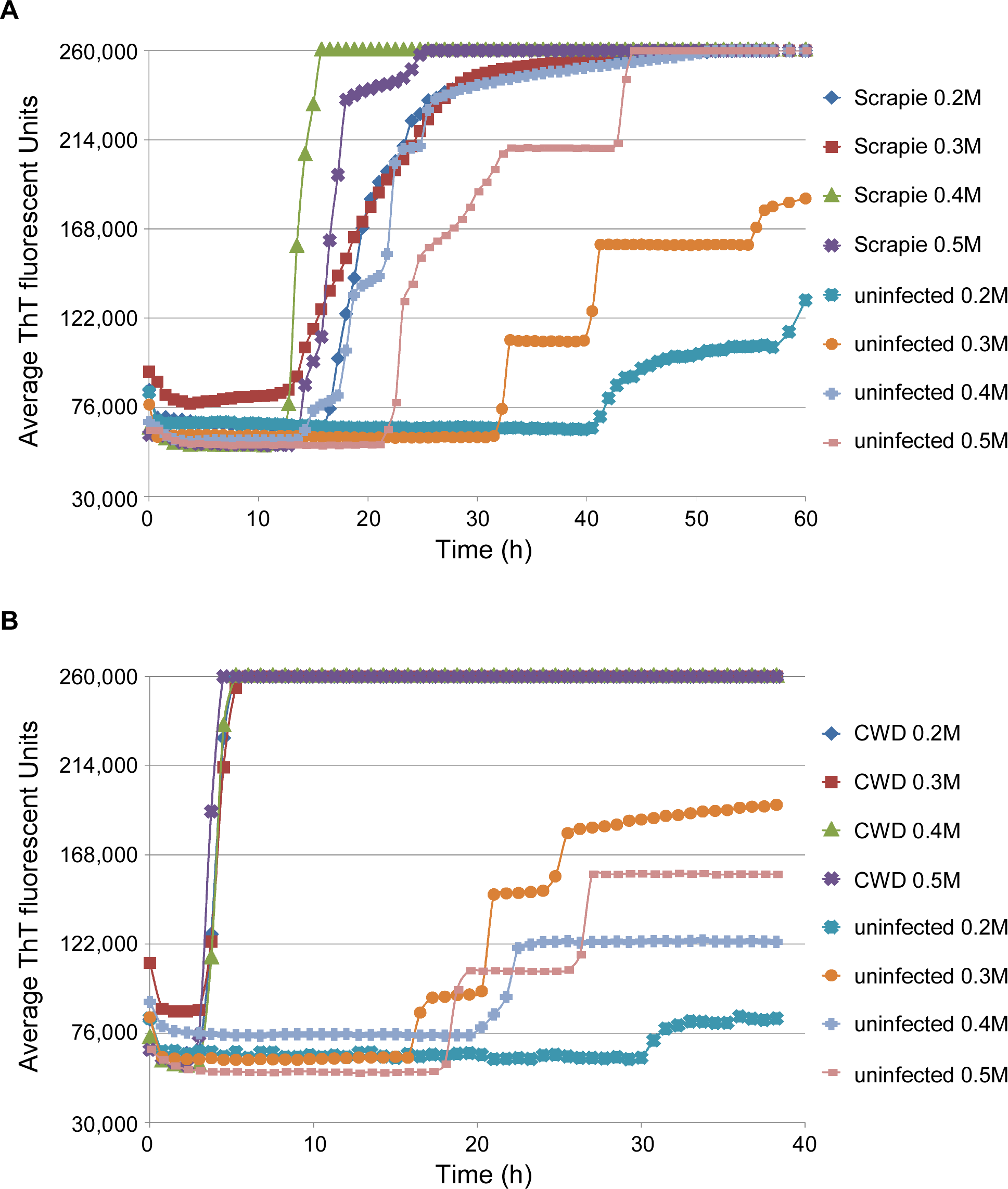

Supplement: Figure S1 — Guanidine HCl effects in RT-QuIC reactions seeded with sheep scrapie and deer CWD. Dilutions of sheep scrapie (10-3.9; ∼100 fg PrPSc) (A) and deer CWD (10-5.3; ∼40 fg PrPCWD) (B) BH's and corresponding dilutions of NBH from the same species were used to seed RT-QuIC reactions. The guanidine-HCl concentration in the reactions was varied as designated. All reactions utilized the homologous full length rPrPC substrates. The data points show the average ThT fluorescence of 4 and 8 replicate wells respectively. (0.24 MB TIF) [file ppat.1001217.s001.tif]
